# Multiple Symbiont Acquisition Strategies as an Adaptive Mechanism in the Coral *Stylophora pistillata*

**DOI:** 10.1371/journal.pone.0059596

**Published:** 2013-03-26

**Authors:** Kristen A. Byler, Maya Carmi-Veal, Maoz Fine, Tamar L. Goulet

**Affiliations:** 1 Department of Biology, University of Mississippi, University, Mississippi, United States of America; 2 Interuniversity Institute of Marine Sciences, Eilat, Israel; 3 Mina and Everard Goodman Faculty of Life Sciences, Bar-Ilan University, Ramat-Gan, Israel; King Abdullah University of Science and Technology, Saudi Arabia

## Abstract

In obligate symbioses, the host’s survival relies on the successful acquisition and maintenance of symbionts. Symbionts can either be transferred from parent to offspring via direct inheritance (vertical transmission) or acquired anew each generation from the environment (horizontal transmission). With vertical symbiont transmission, progeny benefit by not having to search for their obligate symbionts, and, with symbiont inheritance, a mechanism exists for perpetuating advantageous symbionts. But, if the progeny encounter an environment that differs from that of their parent, they may be disadvantaged if the inherited symbionts prove suboptimal. Conversely, while in horizontal symbiont acquisition host survival hinges on an unpredictable symbiont source, an individual host may acquire genetically diverse symbionts well suited to any given environment. In horizontal acquisition, however, a potentially advantageous symbiont will not be transmitted to subsequent generations. Adaptation in obligate symbioses may require mechanisms for both novel symbiont acquisition and symbiont inheritance. Using denaturing-gradient gel electrophoresis and real-time PCR, we identified the dinoflagellate symbionts (genus *Symbiodinium*) hosted by the Red Sea coral *Stylophora pistillata* throughout its ontogenesis and over depth. We present evidence that *S. pistillata* juvenile colonies may utilize both vertical and horizontal symbiont acquisition strategies. By releasing progeny with maternally derived symbionts, that are also capable of subsequent horizontal symbiont acquisition, coral colonies may acquire physiologically advantageous novel symbionts that are then perpetuated via vertical transmission to subsequent generations. With symbiont inheritance, natural selection can act upon the symbiotic variability, providing a mechanism for coral adaptation.

## Introduction

Obligate mutualistic symbioses are ubiquitous on earth and play pivotal roles in many ecosystems [1], [2]. By definition, in obligate mutualisms, the host must possess symbionts in order to survive. If a host secures the perpetuation of obligate symbionts by directly transferring symbionts to the offspring (vertical transmission) [3], progeny encountering an environment that differs from that of their parent may be disadvantaged by hosting a suboptimal symbiont. On the other hand, if a host releases aposymbiotic progeny that must acquire symbionts from the environment (horizontal transmission), progeny may acquire symbionts that are beneficial in a new environment [4]. As partner fidelity is not absolute in horizontal transmission, strong partner choice and symbiont sexual recombination can allow mutualisms to persist and evolve in systems with horizontal transmission [2], [5], [6]. In horizontal transmission however, since subsequent offspring do not inherit the symbionts, advantageous symbionts may or may not be acquired again, leaving each generation to potentially gamble with the continuation of a beneficial symbiosis.

Adaptation, that is maintained via natural selection and subsequent evolution, may be required for species to survive in a changing environment, but neither vertical nor horizontal symbiont acquisition strategies alone provide a mechanism for the adaptation of an obligate symbiosis via symbiont partner change. Vertical transmission provides a means for the perpetuation of symbionts, but offers no mechanism for the acquisition of novel symbionts. In contrast, horizontal transmission provides a mechanism for acquiring novel symbionts but limited means for perpetuating the novel symbionts. Combining both acquisition strategies may provide a mechanism for adaptation, but evidence of the same host species utilizing both transmission modes is rare. Phylogenetic analyses of specific obligate prokaryote-insect [7], [8] and prokaryote-marine invertebrate [9], [10] symbioses reveal predominant vertical symbiont transmission punctuated by infrequent horizontal symbiont acquisition. We investigated whether both symbiont transmission modes could occur in a eukaryote-invertebrate obligate symbiosis.

Reef building corals have an obligatory mutualism with dinoflagellate algae (genus *Symbiodinium*), which provides a nutritional foundation for host metabolism [11] and calcification [12] making them fundamental components of coral reef ecosystems. In the *Symbiodinium* genus, species remain largely unresolved, limiting classification to membership within nine *Symbiodinium* clades (named A–I) and multiple types within each clade [13], [14]. *Symbiodinium* can exhibit different physiologies in response to variations in light and temperature [15]–[17]. Consequently, the same host can display different physiologies based on the *Symbiodinium* found within it [18].

Regardless of whether *Symbiodinium* are acquired horizontally or vertically, adult corals exhibit extremely stable and specific mutualisms with *Symbiodinium* [19]–[24]. In contrast, larvae (planulae) and/or juveniles of coral species with horizontal *Symbiodinium* acquisition can acquire non-parental symbionts [25]–[27]. While the juvenile stage may be key in establishing novel symbioses, no studies to date have demonstrated coral juveniles capable of successfully maintaining a novel symbiont type into adulthood [28], nor have they provided a mechanism for the perpetuation of a novel symbiont to subsequent generations.

Contrary to horizontal symbiont acquisition, vertical transmission is often regarded as a “closed” system that precludes symbiont diversity in all life stages [3], [20], [29]–[32] but see [33]. Regarding vertical symbiont transmission as a closed system may explain why the symbiont identity in planulae and juveniles of coral species with vertical symbiont transmission has not been determined. Only recently has the symbiont identities in eggs of one coral species with vertical symbiont transmission been documented [34]. Planulae and/or juvenile corals with maternally derived symbionts may be capable of subsequent horizontal acquisition, which would facilitate diversity. Importantly, the inheritance of symbionts via vertical transmission would perpetuate the novel symbiosis if it increased the fitness of current and subsequent host generations. Therefore, deciphering the symbiont acquisition strategies utilized by corals throughout ontogenesis is key to understanding corals’ ability, or lack thereof, to alter their symbionts based on the environmental conditions of the habitat in which they grow.

We determined whether horizontal symbiont acquisition could occur in a coral host with vertical symbiont transmission. The coral *Stylophora pistillata* (Pocilloporidae) broods and releases planulae with vertically transmitted *Symbiodinium* [35]. *S. pistillata* is widely distributed throughout the Indo-Pacific and Red Sea [36], and is among the most abundant reef building corals in the Gulf of Eilat, Red Sea [35]. In the Gulf of Eilat, *S. pistillata* adult colonies host two distinct *Symbiodinium* clades. Shallow water colonies (<17 m) associate with clade A *Symbiodinium* [37], [38] (type A1 [39]), while congeners sampled in deep-water harbor symbionts of clade C [38] (e.g. type C72 at 20–30 m [5]).

We examined the *Symbiodinium* genetic identity in *S. pistillata* adults, their released planulae, and juvenile colonies, in shallow and deep depths, using techniques capable of detecting both abundant and possible low-level symbiont populations. We determined whether shallow and deep-water adult *S. pistillata* colonies hosted previously undetected low-levels of the second *Symbiodinium* clade found in *S. pistillata* adults. We also identified the *Symbiodinium* inherited by the planulae. Due to physiological differences between *Symbiodinium,* which symbiont(s) the progeny inherit may affect their survivorship in different habitats [34], [38]. Additionally, we looked at the *Symbiodinium* naturally occurring in juveniles at both shallow and deep depths. If *S. pistillata* juvenile colonies can acquire symbionts from the environment their dual mode of symbiont acquisition may enable rapid adaptation.

## Materials and Methods

### Sample Collection

Samples were collected from a reef in front of the Interuniversity Institute for Marine Sciences in Eilat (IUI), Gulf of Eilat (Aqaba), Red Sea (29° 30′ N, 34° 56′ E). *Stylophora pistillata* colonies were haphazardly collected from both shallow (2–6 m) and deep (24–26 m) water habitats in May, June, and/or July of 2009–2011 using scuba. *S. pistillata* were collected from three distinct age classes: adult colonies (∼15–30 cm width), juveniles colonies (∼0.5–2.8 cm width), and pelagic planulae. Field collection of animals complied with a permit issued by the Israel Nature and National Parks Protection Authority.

From each adult colony, a branch piece of approximately 2 cm in length was collected. Additionally, from each adult colony sampled in 2009 and 2010, spawned planulae were collected using planula collection nets [40]. No planulae were collected in 2011. Entire juvenile *S. pistillata* colonies were haphazardly collected from both depths in July of 2010. All samples were immediately frozen at −80°C or preserved in 95–100% ethanol for DNA analysis.

### DNA Extraction, Amplification, and Denaturing Gradient Gel Electrophoresis

Genomic DNA was extracted from each adult and juvenile coral fragment [39], [41]. Reagent volumes were reduced 10 fold for planula extraction to accommodate the small sample volume [42]. The internal transcribed spacer 2 region of the ribosomal DNA was amplified using the ITSinfor2 and ITS2CLAMP primers developed by LaJeunesse and Trench [43] and amplification conditions described by LaJeunesse [44].

The PCR amplified ITS2 product was electrophoresed on an 8% polyacrylamide denaturing gradient gel (45–80% urea-formamide gradient) at a constant temperature (60°C) for 13 hours at 120 V [20]. Resulting denaturing gradient gel electrophoresis (DGGE) gels were stained with SYBR Green I nucleic acid gel stain (Invitrogen) for at least 20 minutes. Adult corals were run on the same gel with a maximum of 17 of their released planulae. Informative bands were excised [5] and sequenced on an Applied Biosystems 3730 capillary sequencer. For *Symbiodinium* type identification, sequence chromatographs were analyzed manually using Geneious (version 5.3.6) and compared to GenBank submissions.

### Real-time PCR

Real-time PCR was used to evaluate possible low-levels of *Symbiodinium* that fell below the detection limit of DGGE. *S. pistillata* in the Red Sea has only been reported to associate with clade A in shallow water and clade C in deep water [37], [38]. Consequently, clade A (SymA28S) and C (SymC28S) *Symbiodinium* specific primer pairs were used to target the 28S ribosomal region [45].

Prior to running assays, primer optimizations and standard curves were run to confirm optimal primer and DNA concentrations. All samples were run in triplicate on an Applied Biosystems 7300 real-time PCR system. A 25 µl reaction included 0.1–2 ng/µl of DNA, Power SYBR green PCR mastermix (Applied Biosystems), and either 450 nM or 150 nM of clade A or C primers, respectively. Plates were run following standard amplification conditions [46] with a dissociation curve [47]. In addition to positive and negative controls, standard curves were run on each plate in duplicate over five, three-fold serial dilutions from 0.33 ng/µl to 0.004 ng/µl.

In order to accurately compare samples, strict values were set to consistently define a positive reaction. The cycle-threshold (C_T_) represents the PCR cycle at which sample fluorescence surpasses a fixed threshold limit. By comparing the standard curves run on each plate, a fixed threshold value was created based on average automatic software threshold settings for each primer pair [47]. As each primer pair was considered individually, the clade C primer pair had a fixed threshold of 0.57 while the clade A primer pair had a fixed threshold of 1.30.

In addition to a fixed threshold, we determined a cutoff C_T_ value to consistently define a positive versus negative reaction. In competitive mixed clade trials [46], the target clade amplified consistently when it composed at least 0.9% of the total DNA template, setting conservative cutoff C_T_ values of 34 and 32 for the clade A and C primers, respectively. Samples were considered positive if the average C_T_ value was equal to or less than the cutoff C_T_.

## Results

**Figure 1:**
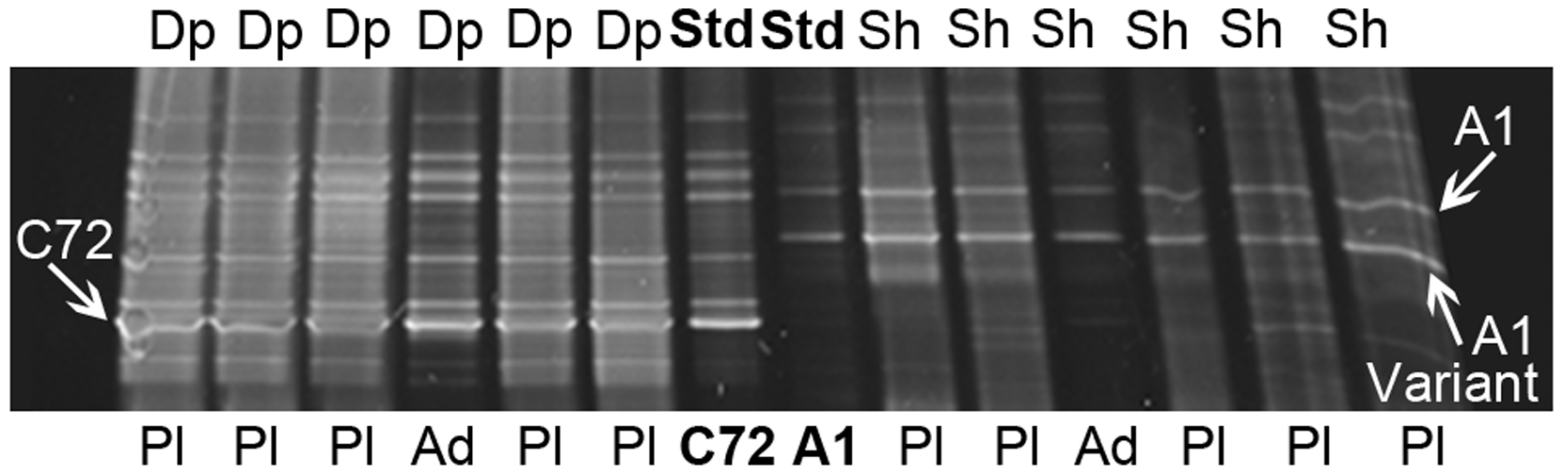
DGGE gel of *Symbiodinium* types present in *Stylophora pistillata* adults (Ad) and released planulae (Pl). Samples were collected from both shallow (Sh, 2–6 m) and deep (Dp, 24–26 m) water. In all cases, the planula DGGE fingerprints were identical to that of their maternal colony. The sequence of the upper dominant band in the DGGE fingerprint from shallow water samples was identical to *Symbiodinium* type A1 (accession AF333505), while the lower dominant band showed a 1 bp difference from A1, indicating *S. pistillata* hosts an A1 variant. All deep-water samples hosted type C72 (accession AY765407). *Symbiodinium* A1 and C72 standards (Std) were run on every gel.

The *Symbiodinium* types present in *S. pistillata* samples collected from shallow (2–6 m) and deep (24–26 m) water habitats were identified using DGGE (Figure 1). Subsequent sequencing of the dominant bands confirmed that all shallow-water adults and their planulae (N = 266) contained *Symbiodinium* type A1. All deep-water adults and their planulae (N = 268) contained *Symbiodinium* type C72 (Figure 1). DGGE, however, cannot detect *Symbiodinium* that constitute less than 5–10% of the total symbiont population [20]. Thus, we also used real-time PCR to detect potential low-level *Symbiodinium* types [45]–[47].

In adult *S. pistillata*, the presence of low-level symbionts varied with depth. Low-level *Symbiodinium* were not detected in any shallow water adult colonies, but low-levels of clade A *Symbiodinium* were detected in some of the deep-water adults analyzed (Table 1). In 2009, all adult deep-water colonies sampled hosted only clade C, but in 2010, the majority of deep-water adult colonies sampled contained low-levels of clade A in addition to the abundant clade C symbionts. In 2011, only one of the sampled deep-water colonies hosted clade A at low-levels (Table 1).

**Table 1:** Abundant and low-level *Symbiodinium* clades in *Stylophora pistillata*.

Year	Depth	Life Stage	N	Abundant Clade	Low-level Clade
2009	Shallow	Adult	10	A (10)	–
		Planula	28	A (28)	–
	Deep	Adult	10	C (10)	–
		Planula	9	C (9)	–
2010	Shallow	Adult	9	A (9)	–
		Planula	134	A (134)	–
		Juvenile	25	A (25)	C (1)
	Deep	Adult	10	C (10)	A (7)
		Planula	107	C (107)	–
		Juvenile	25	C (5); A (11)	–
				A & C (9)*	
2011	Shallow	Adult	10	A (10)	–
	Deep	Adult	10	C (10)	A (1)

*Symbiodinium* clades present in shallow and deep-water *S. pistillata* adults, planulae, and juvenlies analyzed (N) with real time PCR. Numbers in parentheses denote the number of samples containing a given clade.

* Samples that contained both A and C at levels detectable by DGGE, hence both clades were listed as abundant.

All planulae processed, from both depths, hosted a single *Symbiodinium* clade with no detectable low-levels of the second clade (Table 1). Even maternal colonies hosting both abundant and low-level *Symbiodinium* released planulae without the low-level symbiont. Using one-sample proportion tests, we tested a series of statistical null hypotheses to determine the lowest proportion of released planulae that may contain low-level *Symbiodinium* to determine if the lack of detection of additional symbionts was due to under-sampling. We were able to reject the null hypothesis that ≥2% of planulae released from shallow water adults may contain low-levels of clade C *Symbiodinium* (N = 161, df = 1, χ^2^ = 3.286, p-value = 0.035). Similarly, we rejected the null hypothesis that ≥3% of planulae released from deep-water adults may contain low-levels of clade A *Symbiodinium* (N = 116, df = 1, χ^2^ = 3.588, p-value = 0.029). Consequently, our sampling of planulae is most likely representative of what naturally occurs on the reef. Furthermore, based on the DGGE analysis, none of the planulae analyzed had a symbiont composition that differed from their parent.

Some juvenile *S. pistillata* colonies harbored a mixture of symbionts, in stark contrast to planulae, which only contained the abundant parental symbiont clade. Similar to the results for shallow water adult colonies, the majority (24/25) of juveniles collected in shallow water hosted only clade A *Symbiodinium*. One juvenile collected in shallow water, however, hosted both clade A and low-levels of C simultaneously. Of the 25 juveniles collected in deep-water, only five individuals solely contained clade C *Symbiodinium*. Nine other deep-water juveniles hosted both clades C and A *Symbiodinium* (Table 1). Eleven of the deep-water juveniles analyzed in the present study solely contained clade A *Symbiodinium*, with no detectable traces of clade C (Table 1), which is the dominant symbiont hosted by deep-water adult colonies.

## Discussion

In obligate symbioses, in which the symbiont is vertically transmitted from parent to offspring, the offspring are guaranteed to receive the obligate symbiont. Vertical symbiont transmission in itself, however, is not necessarily straightforward. If different parents within a species host different symbionts, or if the same parent hosts multiple genetically distinct symbionts, progeny may inherit all or any one of the symbionts. In the case of *Stylophora pistillata*, we detected low-level symbionts in some adult colonies, indicating the potential for diverse *Symbiodinium* combinations in planulae. Which symbiont(s) the offspring inherit may vary between and even within a single parent. In addition, if progeny that inherit symbionts can later acquire symbionts from the environment, the symbiont variation within the coral species may further increase. If the symbionts differ physiologically, then which of the various symbionts the progeny acquire (either from a parental or environmental source) may affect their fitness.

The *Symbiodinium* genetic identity in coral species with vertical symbiont transmission has not been investigated extensively and we are aware of only one study that determined the *Symbiodinium* inherited in eggs of the coral *Montipora capitata* in Hawai’i [34]. While only approximately 35% of coral species vertically transmit *Symbiodinium*, these coral species belong to several widely distributed, dominant coral genera, e.g. *Porites*, *Montipora*, and *Pocillopora* [48]. Consequently, investigating the symbiont identity throughout ontogenesis in the numerous ecologically dominant coral species with vertical symbiont transmission is ecologically relevant to understanding coral–algal symbioses and coral reefs in general. It is equally important to determine if species with vertical *Symbiodinium* transmission are capable of symbiont acquisition from the environment.

Our results corroborate previous *Symbiodinium* identification in adult *S. pistillata* in the Gulf of Eilat, whereby adult *S. pistillata* colonies host two different *Symbiodinium* clades as a function of depth [37], [38]. Several coral species, with horizontal or vertical symbiont transmission, host different *Symbiodinium*, either at the same depth or over a depth gradient [15], [49], [50]. By employing molecular techniques with finer resolution, it has been demonstrated that, in some of these species, colonies can host one *Symbiodinium* type at abundant levels, in addition to a second symbiont type present at low-levels [51]. Using real-time PCR, we uncovered that the *S. pistillata* adult colonies sampled in shallow water only hosted clade A *Symbiodinium,* while colonies in deep-water could harbor low-levels of clade A in addition to the abundant levels of clade C *Symbiodinium*.

The symbiont depth zonation observed in *S. pistillata* in the Gulf of Eilat, at both abundant and low-levels, may be due to symbiont niche partitioning [4]. Clades A and C *Symbiodinium* hosted by adult *S. pistillata* colonies display differential responses to both elevated temperature and irradiance [38], [52]. Adult colonies hosting clade C are less resilient to thermal stress than colonies hosting clade A [38]. Additionally, cell size and chlorophyll content differ between *Symbiodinium* types A1 and C72 hosted by shallow and deep-water *S. pistillata* colonies, respectively [38]. These physiological differences may explain why in the present study, type C72 *Symbiodinium* appears mostly limited to *S. pistillata* in deeper water while type A1 is capable of surviving in colonies at both depths. The presence of clade A *Symbiodinium* in deep-water adults varied from individual to individual. Since we did not repeatedly sample the same colonies, we cannot ascertain whether low-levels of clade A are hosted permanently or transiently in some deep-water adult colonies. Hosting transient symbionts at abundant and low-levels, even those not known to associate with a given host, has been documented in temporal studies [20], [46], [53].

Regardless of whether *S. pistillata* maternal colonies hosted only one *Symbiodinium* clade, or had low-levels of the other *Symbiodinium* clade, all the planulae, from both depths, were released with only the abundant maternal *Symbiodinium* type. On the other hand, Padilla-Gomiño *et al.* [34] reported several instances in which *Montipora capitata* eggs harbored both parental and non-parental symbiont types. Although the techniques employed by Padilla-Gomiño *et al.* [34] may overestimate symbiont diversity [54], clade level differences were noted, with some of the non-parental symbiont clades and types known to associate with *M. capitata* in Hawai’i. As *M. capitata* vertically transmits *Symbiodinium*, the authors raised three hypotheses to explain these results, including environmental contamination, sampling bias, and the potentiality of horizontal symbiont acquisition in *M. capitata* eggs [34].

**Figure 2:**
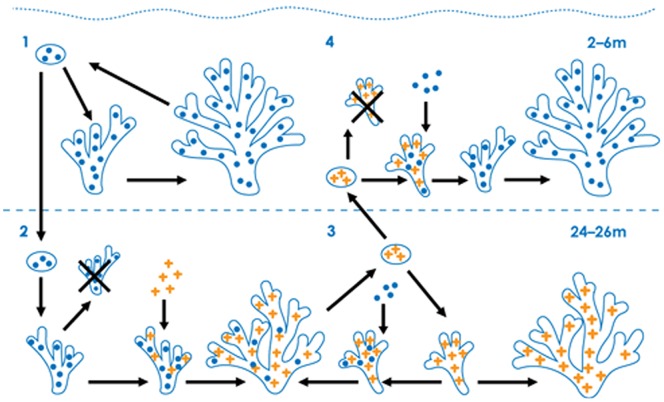
Schematic depicting potential scenarios (1–4) of symbiont inheritance and acquisition throughout *Stylophora pistillata* ontogenesis. (1) Shallow water adults and planulae only host clade A *Symbiodinium* (•). Planulae settling in shallow water will become adults hosting clade A. (2) Some planulae from shallow water adults may settle in deep-water. These juveniles may horizontally acquire clade C *Symbiodinium* (+) while juveniles hosting only clade A may perish (×). (3) Deep-water adults abundantly host clade C *Symbiodinium* (potentially low-levels of clade A), and planulae only inherit clade C. Upon settlement in deep-water, juveniles will maintain clade C or horizontally acquire clade A. (4) Planulae from deep-water adults may settle in shallow water. Juveniles only hosting clade C may perish; horizontally acquiring clade A may facilitate survival to adulthood.

Our study provides evidence for the possibility of horizontal symbiont acquisition in a species with vertical symbiont transmission since *S. pistillata* planulae contained only one *Symbiodinium* type while some of the juvenile colonies, at both shallow and deep depths, harbored mixed symbioses. The incongruity between the symbionts in adult *S. pistillata,* the planulae and juveniles is likely explained by events occurring during the juvenile phase, and we present four plausible scenarios that may lead to the symbiont depth zonation observed in adult *S. pistillata* colonies. For example, if planulae released from shallow water colonies, with their clade A *Symbiodinium* complement, settle and metamorphose in deep-water, the resulting juveniles will initially contain clade A *Symbiodinium* (Figure 2). Two possible, not mutually exclusive, scenarios may then follow, both leading to the observed adult *S. pistillata* symbiosis with abundant clade C *Symbiodinium* in deep-water. First, juveniles that continue to maintain only clade A *Symbiodinium* may not survive to adulthood (Figure 2). Alternatively, if clade A containing juveniles in deep-water horizontally acquire clade C *Symbiodinium*, clade C may outcompete clade A and become the abundant symbiont present in adulthood (Figure 2). We may have witnessed a snapshot of this process in the nine juveniles that contained both *Symbiodinium* clades simultaneously. Similar scenarios may apply to shallow water *S. pistillata* (Figure 2).

Horizontal symbiont acquisition in the juvenile phase offers the most parsimonious explanation for the presence of multiple *Symbiodinium* clades within several juvenile colonies and the lack of multiple symbiont clades within all planulae analyzed. Alternatively, very low-levels of background symbionts could be present in some planulae, which could explain the presence of multiple symbiont clades in some juveniles. The real-time PCR assay reliably detected a *Symbiodinium* clade comprising at least 1% of the total symbiont population. Given the large volume of planulae released from a single parental colony over the course of the spawning season, it is possible that a small number of planulae inherit multiple symbionts, although statistical analyses indicated that this is very unlikely (see results). Given the tools utilized, for this coral species, horizontal symbiont acquisition in addition to vertical symbiont transmission seems plausible, although it remains to be determined whether juveniles that acquire symbionts from the environment survive to adulthood.

The occurrence of both vertical and horizontal symbiont transmission modes within a single host species, although not necessarily within a single individual, has previously been inferred in studies on vertically transmitted prokaryotic symbionts. These studies detected phylogenetic evidence of horizontal symbiont transmission [7]–[10], [55], although the frequency and life stage of acquisition were not determined. By sampling members of a coral species throughout its ontogeny, we were able to investigate a mutualism with inherited eukaryotic symbionts that may also engage in horizontal symbiont acquisition.

### Conclusions

The ecological and evolutionary implications of employing both modes of symbiont transmission are substantial. Horizontal acquisition of novel symbionts may be a means by which coral species can adapt to environmental changes [56]. Although sexual reproduction in *Symbiodinium* occurs relatively infrequently [39], [57], [58], new host-symbiont combinations can emerge that may lead to novel, advantageous, and potentially specific symbioses [5]. Horizontal symbiont acquisition in adult corals, however, may either not occur or may be transient [19], [20], [41], [46], [59]. In coral species with horizontal symbiont acquisition, the juvenile stage appears to be more flexible in acquiring symbionts not present in the adult population [25]–[28], [42], [60]. Thus, horizontal symbiont acquisition may allow an individual juvenile to obtain, and subsequently maintain, novel symbionts. In turn, this may increase the juvenile’s fitness and survival as it grows into an adult coral, potentially enabling short-term acclimation on an individual level.

Horizontal symbiont acquisition, however, does not provide a mechanism for the perpetuation of the novel symbionts in subsequent generations. Since symbionts are not transferred to the progeny, the advantageous symbionts will be lost each generation when the progeny must acquire symbionts anew. In contrast, if juvenile corals with vertically transmitted symbionts are capable of acquiring novel, advantageous *Symbiodinium* that are maintained into adulthood, subsequent vertical symbiont inheritance to their progeny would facilitate the maintenance of novel *Symbiodinium* over generations.

Vertical symbiont inheritance, punctuated with horizontal symbiont acquisition, provides an evolutionary mechanism for adaptation to environmental changes through the acquisition and maintenance of advantageous symbionts. *S. pistillata*, for example, may benefit from both modes of symbiont transmission. On the one hand, the progeny are equipped with inherited *Symbiodinium*, eliminating the risk of not obtaining their obligate symbionts. On the other hand, juveniles may acquire novel symbionts, potentially increasing their chances of survival in a new environment. If the horizontally acquired novel symbionts improve host fitness, and become abundant in the resulting adult coral, they will be transferred to the brooded progeny, thereby perpetuating the novel symbiosis. Natural selection can then act on the genetic variation in the symbiosis, potentially establishing novel host-symbiont combinations that may be advantageous during changing environmental conditions.
